# Genetic polymorphisms in the *C19orf66* gene influenced HIV-1 infection in a Yunnan population

**DOI:** 10.7717/peerj.16005

**Published:** 2023-09-07

**Authors:** Yaxiang Zhang, Yue Feng, Yang Liu, Li Liu, Xueshan Xia, A-Mei Zhang

**Affiliations:** 1 Kunming University of Science and Technology, Kunming, China; 2 Kunming Medical University, Kunming, China

**Keywords:** HIV-1 infection, The C19orf66 gene, SNP, Biochemical indices, Subtype

## Abstract

**Background:**

Due to the deficiencies of vaccines and effective medicine, the human immunodeficiency virus (HIV) infection mechanism should be studied. The *C19orf66* gene, one of the interferon-stimulated genes (ISGs), expresses broad-spectra anti-viral activity, including inhibiting HIV replication.

**Methods:**

In this study, we collect 421 HIV-1 infected patients and 448 controls to genotype three SNPs in the *C19orf66* gene. Then, the association between SNPs and biochemical indices/ HIV-1 subtypes are analyzed.

**Results:**

Genotypes CC and CT of rs12611087 show statistically lower and higher frequencies in HIV-1 infected patients than in controls, respectively. Alleles C and T of rs12611087 play protective and risk roles in Yunnan HIV population, respectively. Biochemical indices analysis shows that HIV-1 infected persons carried genotype TT of rs77076061 express significantly lower CD3^+^/CD45^+^ ratio level and higher IBIL level. The epidemic subtypes of HIV-1 patients in this study are CRF 07_BC and CRF 08_BC. Moreover, subtype CRF 08_BC tends to infect persons with genotype CC of rs12611087.

**Conclusion:**

The genetic polymorphisms of the *C19orf66* gene are firstly studied and reported to associate with HIV-1 infection and biochemical indices of patients in Yunnan. Furthermore, subtype CRF 08_BC infection could be influenced by genotypes of SNP in the *C19orf66* gene.

## Introduction

Human immunodeficiency virus (HIV) infection damages human’s immunity and leads to Acquired Immune Deficiency Syndrome (AIDS). Some opportunistic pathogens (including tuberculosis, Kaposi’s sarcoma-associated herpesvirus (KSHV), *etc*.) will invade into host and lead to serious infectious disease or cancers after HIV-1 infection. In 2020, the numbers of HIV infected persons reached to 38 million, and it might reach to 42 million persons infected with HIV in 2030 (Dybul et al., 2021). Although some medicines have been used to treat HIV infected individuals, no vaccine and medicine could effectively protect persons from HIV infection. Thus, further investigating mechanisms of HIV infection and pathogenesis is necessary.

Host genetic and immune factors are identified to participate into antiviral responses. Virus infection reduces interferon level, which limits viral replication by pulse-on expression of the interferon-stimulated genes (ISGs). Interestingly, the *C19orf66* gene, encoded a 291 amino acid protein, was firstly investigated and considered as an ISG in 2011 (Schoggins et al., 2011). In recent ten years, C19orf66 was reported to be a broad-spectrum anti-viral factor and inhibit HIV, Hepatitis C virus (HCV), Zika virus, and so forth (Wang et al., 2019; Kinast et al., 2020; Wu et al., 2020). C19orf66 inhibits Gag protein expression of HIV by programmed-1ribosomal frameshifting (-1PRF) (Wang et al., 2019). Host genetic polymorphisms play important roles in regulating protein function. Thus, analyzing genetic polymorphisms of the *C19orf66* gene in HIV-1 patients are necessary.

In this study, HIV-1 patients and controls are collected and analyzed to investigate the relationship between genetic polymorphisms of the *C19orf66* gene and HIV infection, biochemical indices of patients, and subtypes of HIV-1.

## Materials and Methods

### Individuals

A total of 421 HIV-1 patients and 448 general controls are collected from Yunnan Province. Patients are identified as HIV-1 infection by using Anti-HIV ELISA Kit (Wantai, Beijing, China), who are not treated by any medicines when information was collection. All patients are not carried with Hepatitis B virus and HCV infection after detection by using Quantitative CLIA Kit (Autobio, Zhengzhou, China) and HCV ELISA Kit (ORTHO, USA). Controls are gender-, age-, and region- matched persons, who are detected and identified without HBV, HCV, and HIV infection. All individuals are collected three mL whole blood and parameters of blood routine and liver function test. The parameters of liver function test included alanine transaminease (ALT), aspartate transaminase (AST), total bilirubin (TBIL), direct bilirubin (DBIL), indirect bilirubin (IBIL), total protein (TP), albumin (ALB), and globin (GLOB). The parameters of blood routine included white blood cells (WBC), neutrophilic granulocyte (NEUT), lymphocytes (LYM), monocytes (MONO), eosinophil granulocyte (EO), basophile granulocyte (BASO). At the same time, the numbers of cells expressed CD3^+^, CD4^+^, CD8^+^, and CD45^+^ of HIV-1 infected persons are detected for further analysis. The results of these tests are presented by mean ± SE in each group. Written informed consent conforming to the tenets of the Declaration of Helsinki was obtained from each participant prior to the study. This study was approved by the Institutional Review Board of Kunming University of Science and Technology (Approval No. KMUST-MEC-054).

### Single nucleotide polymorphisms (SNPs) selection and genotyping

Genomic DNA is extracted from 200 µL whole blood cells of each individual by using RelaxGene Blood DNA system (Tiangen, Beijing, China). Three tag single nucleotide polymorphisms (SNPs, rs77076061, rs1979262, and rs12611087) in the *C19orf66* gene are selected for further genotyping (by using SNPinfo). Genotypes and alleles are analyzed by using SNapShot assay and identified in 10% samples by using sequencing.

### HIV-1 genotypes

HIV RNA is extracted from serum of each HIV-1 infected person by using TIANamp Virus RNA Kit (Tiangen, Beijing, China). Then, the pol region (about 1.4 kb length) is amplified by using methods in our previous study (Zhang et al., 2019). HIV sequences are analyzed and genotyped by using COMET HIV-1 (https://comet.lih.lu/index.php?cat=hiv1) (Struck et al., 2014).

### Statistical analysis

The Hardy–Weinberg equilibrium (HWE) was assessed for each SNP to identify the deviation. Genotype and allele frequencies of each SNP are compared between HIV patients and controls by using the Pearson Chi-square test. The parameters of liver function test and blood routine are compared between HIV patients and controls by using student *t* test (two-tailed). The parameters of liver function test and blood routine of HIV patients with different genotypes of each SNP are analyzed by using student *t* test (two-tailed). Genotype frequencies of each SNP in patients with different subtypes are analyzed by using the Pearson Chi-square test. The GPower 3.1.9.7 software was used to calculate the powers of samples. GraphPad Prism 8.2.1 software (GraphPad Software, Inc., San Diego, CA, USA) is used and it is considered significant difference when the *P*-value is less than 0.05.

## Results

The mean age of HIV patients and controls are 40.28 ± 0.70 and 40.58 ± 0.53, respectively. 297 males and 124 females are HIV patients, and controls include 275 males and 173 females. Most parameters of blood routine and liver function test show significant difference between HIV-1 patients and controls. However, ALT, AST, DBIL, and MONO levels are similar between HIV-1 patients and controls (Table 1).

**Table 1:** Comparison of information between HIV patients and controls.

		HIV patients	Controls (*N* = 448)	*P*-value
Gender	Male	297	275	>0.05
	Female	124	173	
Age		40.28 ± 0.70	40.58 ± 0.53	>0.05
viral load (log10)		4.69 ± 0.07	–	–
AST (5–40 U/L)		39.07 ± 8.29	24.63 ± 0.53	0.066
ALT (5–40 U/L)		26.42 ± 1.02	29.00 ± 1.04	0.079
TBIL (3.4–20.5 µmol/L)		13.80 ± 0.56	11.66 ± 0.28	0.0004
DBIL (0–6.8 µmol/L)		4.46 ± 0.35	3.86 ± 0.09	0.083
IBIL (0–13.7 µmol/L)		9.34 ± 0.26	7.78 ± 0.19	<0.0001
TP (65–80 g/L)		76.46 ± 0.37	78.57 ± 0.20	<0.0001
ALB (35–55 g/L)		43.41 ± 0.31	47.18 ± 0.13	<0.0001
GOLB (20–30 g/L)		33.24 ± 0.36	31.53 ± 0.18	<0.0001
WBC ([4.0–10.0] × 10^9^/L)		5.40 ± 0.09	6.70 ± 0.12	<0.0001
NEUT ([2.0–7.5] × 10^9^/L)		3.19 ± 0.08	3.77 ± 0.06	<0.0001
LYM ([0.8–4.0] × 10^9^/L)		1.66 ± 0.04	2.25 ± 0.03	<0.0001
MONO ([0.12–0.8] × 10^9^/L)		0.42 ± 0.01	0.57 ± 0.11	0.203
EO ([0.02–0.5] × 10^9^/L)		0.11 ± 0.01	0.14 ± 0.005	0.001
BASO ([0–0.1] × 10^9^/L)		0.03 ± 0.001	0.03 ± 0.001	0.001

**Notes.**

Gender and age are analyzed in 421 HIV patients; the biochemical characteristics data are analyzed in 391 HIV patients; viral loads are analyzed in 247 HIV patients.

ALT alanine transaminease AST aspartate transaminase TBIL total bilirubin DBIL direct bilirubin IBIL indirect bilirubin TP total protein ALB albumin GLOB globin WBC white blood cells NEUT neutrophilic granulocyte LYM lymphocytes MONO monocytes EO eosinophil granulocyte BASO basophile granulocyte

The HWE results suggest no deviation was found in each SNP of HIV patients and controls. Furthermore, the power of sample size in this study is more than 0.9 in both HIV patients and controls, respectively. The frequencies of genotypes and alleles of rs12611087 show statistical difference between HIV patients and controls (Table 2). Genotype CC shows a higher frequency in controls (77.45%, 347/448) than in HIV-1 patients (68.88%, 290/421), and *P*-value is 0.006. However, the frequency of genotype CT is significantly lower in controls (20.53%, 92/448) than in HIV patients (27.79%, 117/421) (*P* = 0.016). Allele C of rs12611087 is protective factor for HIV-1 infection in Yunnan individuals. The frequency of allele C is higher in controls (87.82%, 786/896) than in HIV-1 patients (85.78%, 697/842) (*P* = 0.005). Allele T plays risk role in Yunnan population for HIV-1 infection.

**Table 2:** Analysis of genotypes and alleles in the C19orf66 gene between HIV infected persons and controls.

SNP	HIV patients (*N* = 421)	Controls (*N* = 448)	*P*-value	OR (95% CI)
rs77076061	HWE ^*^P = 0.40	HWE *P* = 0.84	
Genotype AA	2	4	0.739	0.530 (0.100–2.284)
AT	84	66	0.052	1.443 (1.011–20.47)
TT	335	378	0.079	0.721 (0.507–1.018)
Allele A	88	74	0.137	1.296 (0.934–1.788)
T	754	822		0.771 (0.559–1.070)
rs1979262	HWE *P* = 0.93	HWE *P* = 0.15	
Genotype AA	2	3	0.944	0.708 (0.125–3.484)
AG	47	38	0.224	1.356 (0.869–2.100)
GG	372	407	0.275	0.764 (0.494–1.197)
Allele A	51	44	0.345	1.248 (0.835–1.890)
G	791	852		0.801 (0.529–1.198)
rs12611087	HWE *P* = 0.87	HWE *P* = 0.62	
Genotype CC	290	347	0.006	0.644 (0.476–0.877)
CT	117	92	0.016	1.489 (1.085–2.032)
TT	14	9	0.319	1.678 (0.740–4.073)
Allele C	697	786	0.005	0.673 (0.515–0.880)
T	145	110		1.487 (1.137–1.940)

**Notes.**

* Chi-square test for deviation from the Hardy–Weinberg equilibrium (a value of *P* < 0.05 was regarded as a deviation from the HWE).

In order to investigate whether biochemical parameters are associated with genotypes of SNPs in the *C19orf66* gene, HIV patients are divided into two groups due to less numbers of genotypes of each SNP. The CD3^+^/CD45^+^ ratio significantly increased in HIV patients with genotype AA and AT of rs77076061 (100.3 ± 25.50%) than in patients with genotype TT (75.43 ± 0.7%, *P* = 0.041). IBIL level is much higher in HIV patients with genotype AA and AT (11.01 ± 1.21) than in patients with genotype TT (9.15 ± 0.25, *P* = 0.018) (Table 3).

**Table 3:** Analysis of biochemical indices among HIV patients carried various genotypes of SNPs in the C19orf66 gene.

Marker	rs77076061			Marker	rs1979262		
	AA & AT (*n* = 86)	TT (*n* = 335)	*P*- value		AA & AG (*n* = 49)	GG (*n* = 372)	*P*- value
RNA (log10 copies)	4.63 ± 0.13	4.71 ± 0.08	0.660	RNA (log10 copies)	4.55 ± 0.17	4.71 ± 0.07	0.439
CD3^+^/CD45^+^(40–85%)	100.3 ± 25.50	75.43 ± 0.70	0.041	CD3^+^/CD45^+^(40–85%)	75.33 ± 1.70	80.52 ± 5.24	0.738
CD3^+^ (955–2860 cells/µL)	1181 ± 73.32	1,276 ± 38.38	0.279	CD3^+^ (955–2,860 cells/µL)	1,144 ± 95.60	1,272 ± 26.39	0.257
CD3^+^&CD4^+^/CD45^+^(30–54%)	18.71 ± 1.59	17.40 ± 0.61	0.378	CD3^+^&CD4^+^/CD45^+^(30–54%)	15.93 ± 1.87	17.84 ± 0.60	0.313
CD3^+^&CD4^+^(706–1,125 cells/µL)	281.8 ± 27.02	295.9 ± 13.21	0.646	CD3^+^&CD4^+^(706–1,125 cells/µL)	253.3 ± 33.37	297.9 ± 12.64	0.254
CD3^+^&CD8^+^/CD45^+^(15–34%)	51.78 ± 1.63	53.04 ± 0.89	0.533	CD3^+^&CD8^+^/CD45^+^(15–34%)	53.72 ± 2.08	52.71 ± 0.84	0.695
CD3^+^&CD8^+^ (323-836 cells/µL)	800.4 ± 59.89	890.1 ± 31.21	0.213	CD3^+^&CD8^+^ (323–836 cells/µL)	815.5 ± 77.37	880.3 ± 29.69	0.480
CD4^+^/CD8^+^(1–2.87)	0.39 ± 0.04	0.41 ± 0.02	0.833	CD4^+^/CD8^+^(1–2.87)	0.36 ± 0.07	0.41 ± 0.02	0.379
TBIL (3.4–20.5 µmol/L)	15.74 ± 2.44	13.33 ± 0.36	0.086	TBIL (3.4–20.5 µmol/L)	13.57 ± 1.17	13.82 ± 0.61	0.887
DBIL (0–6.8 µmol/L)	5.66 ± 1.71	4.16 ± 0.14	0.094	DBIL (0-6.8 µmol/L)	4.63 ± 0.73	4.44 ± 0.39	0.869
IBIL (0–13.7 µmol/L)	11.01 ± 1.21	9.15 ± 0.25	0.018	IBIL (0–13.7 µmol/L)	8.94 ± 0.55	9.39 ± 0.28	0.596
TP (65–80 g/L)	75.04 ± 0.91	76.81 ± 0.40	0.057	TP (65–80 g/L)	77.02 ± 0.88	76.40 ± 0.40	0.604
ALB (35-55 g/L)	42.82 ± 0.73	43.55 ± 0.34	0.339	ALB (35–55 g/L)	43.78 ± 0.92	43.36 ± 0.32	0.674
GOLB (20–30 g/L)	32.23 ± 0.79	33.49 ± 0.40	0.160	GOLB (20–30 g/L)	33.24 ± 1.33	33.24 ± 0.37	0.998
AST (5–40 U/L)	29.56 ± 1.46	41.42 ± 10.33	0.570	AST (5–40 U/L)	39.26 ± 6.44	39.05 ± 9.27	0.994
ALT (5–40 U/L)	25.18 ± 1.88	26.72 ± 1.18	0.547	ALT (5–40 U/L)	26.98 ± 2.46	26.35 ± 1.10	0.848
	rs12611087						
	CC (*n* = 290)	CT & TT (*n* = 131)	*P*- value				
RNA (log10 copies)	4.72 ± 0.08	4.62 ± 0.13	0.538				
CD3^+^/CD45^+^(40–85%)	81.49 ± 6.50	76.07 ± 1.00	0.608				
CD3^+^ (955–2,860 cells/µL)	1,237 ± 39.85	1316 ± 65.98	0.302				
CD3^+^&CD4^+^/CD45^+^(30–54%)	18.20 ± 0.71	16.17 ± 0.92	0.115				
CD3^+^&CD4^+^(706–1,125 cells/µL)	298.5 ± 14.21	279.8 ± 21.44	0.483				
CD3^+^&CD8^+^/CD45^+^(15–34%)	52.49 ± 0.94	53.64 ± 1.43	0.513				
CD3^+^&CD8^+^ (323–836 cells/µL)	850.3 ± 32.07	934.7 ± 55.06	0.175				
CD4^+^/CD8^+^(1-2.87)	0.42 ± 0.02	0.38 ± 0.04	0.339				
TBIL (3.4-20.5 µmol/L)	13.87 ± 0.75	13.64 ± 0.62	0.836				
DBIL (0–6.8 µmol/L)	4.50 ± 0.49	4.36 ± 0.30	0.858				
IBIL (0–13.7 µmol/L)	9.38 ± 0.33	9.26 ± 0.40	0.840				
TP (65–80 g/L)	76.48 ± 0.43	76.42 ± 0.73	0.936				
ALB (35–55 g/L)	43.14 ± 0.36	44.02 ± 0.57	0.189				
GOLB (20–30 g/L)	33.46 ± 0.42	32.74 ± 0.67	0.350				
AST (5–40 U/L)	41.81 ± 11.85	32.79 ± 2.70	0.618				
ALT (5–40 U/L)	26.61 ± 1.28	25.97 ± 1.59	0.770				

**Notes.**

The viral loads are analyzed in 247 HIV patients; the liver function and blood routine data are analyzed in 391 HIV patients; numbers of T cells with different antigen are analyzed in 311 HIV patients.

ALT alanine transaminease AST aspartate transaminase TBIL total bilirubin DBIL direct bilirubin IBIL indirect bilirubin TP total protein ALB albumin GLOB globin WBC white blood cells NEUT neutrophilic granulocyte LYM lymphocytes MONO monocytes EO eosinophil granulocyte BASO basophile granulocyte CD3 ^+^, CD4^+^, CD8^+^, and CD45^+^ cells contained corresponding antigen

HIV-1 subtypes are successfully obtained from 325 HIV-1 infected patients. Eight sub-genotypes are identified in these patients, including CRF 01_AE, CRF 07_BC, CRF 08_BC, 5501B, CRF 64_BC, CRF 85_BC, B, and C. The subtype CRF 07_BC (*n* = 122, 37.54%) and CRF 08_BC (*n* = 119, 36.62%) are domain prevalence in Yunnan. 59, 3, 2, 4, 6, and 10 persons belong to subtype CRF 01_AE, 5501B, CRF 64_BC, CRF 85_BC, B, and C, respectively.

The association between SNP genotypes and HIV-1 subtypes is analyzed. The results suggest genotypes of rs12611087 could influence the infection of HIV-1 patients with subtype CRF 08_BC (Table 4). The genotype frequencies significantly differ among patients infected with subtype CRF 08_BC (*P* = 0.014). Compared to other HIV-1 subtypes, CRF 08_BC prefers to infect persons with genotype CC of rs12611087 than those with genotypes CT and TT of rs12611087 (66.39%, *P* = 0.003). The genotype CT of rs12611087 plays protected role in CRF 08_BC infection (19.33%, *P* = 0.006). No significant difference is identified between the other two SNPs and HIV-1 subtypes.

**Table 4:** Frequency of SNP genotypes in 325 HIV patients with different viral subtype.

SNP/HIV genotype	CRF 01_AE	CRF 07_BC	CRF 08_BC	5501B	CRF 64_BC	CRF 85_BC	B	C	Total
rs7776061									
AA& AT	8	20	26	1	1	0	1	0	57
TT	51	102	93	2	1	4	5	10	268
*P*-value	0.484	0.787	0.161	0.440	0.321	0.999	0.999	0.220	–
rs1979262									
AA&AG	9	15	12	1	1	1	1	0	40
GG	50	107	107	2	1	3	5	10	285
*P*-value	0.588	0.866	0.452	0.327	0.231	0.410	0.548	0.618	–
rs12611087									
CC	32	79	91	2	1	3	5	7	220
CT	24	41	23	1	1	1	1	2	94
TT	3	2	5	0	0	0	0	1	11
*P*-value	0.050	0.175	0.014	0.940	0.791	0.909	0.689	0.444	–

**Notes.**

CRF 01_AE, CRF 07_BC, CRF 08_BC, 5501B, CRF 64_BC, CRF 85_BC, B, and C means the HIV subtypes. The numbers of patients with various genotypes of each SNP were compared in each HIV subtypes.

## Discussion

HIV belongs to *Retroviridae family*, which infection could impair immune system of host. HIV-1 is the epidemic strain worldwide, while HIV-2 is mainly infected to persons in Western and Central Africa (Fanales-Belasio et al., 2010). Until now, about 80 million persons have been infected with HIV-1 and half of them died of AIDS or related diseases (Nchioua et al., 2020). The host innate immune is considered as important antiviral factors for HIV infection (Jia, Zhao & Xiong, 2015), which is associated with genetic polymorphisms. However, HIV could escape from the supervision of host immunity. Thus, study of genetic variations in host immune system could further investigate the HIV pathogenesis.

Although most of the biochemical indices showed significantly difference between HIV patients and controls, all of them ranged in the reference value. These results suggest that HIV infection did not significantly change the biochemical indices of patients. HIV infection could impair CD4^+^ T cells and weaken human immunity system, which increases the infectious risk of opportunistic pathogenic bacteria (WHO, 2022). HIV-1 could persistently exist in infected CD4^+^ T cells and cause to ineffective treatment. CD4^+^ T cells are positively correlated with serum IFN-*γ* level of HIV patients, and is negative correlated with HIV RNA level. This suggests IFN-*γ* might play a distinct role in HIV infection (Okay et al., 2020). Type I IFNs play pivotally restricting roles in acute HIV infection by activating the transcription of hundreds of ISGs (Doyle, Goujon & Malim, 2015). ISGs are considered as potential therapeutic target for HIV infected persons (Bourke et al., 2018). The level of interferon stimulated gene 15 (ISG15) correlates with trail and IDO in HIV-1 viremic patients, but no influence is identified between SNPs and ISG15 levels (Scagnolari et al., 2016). However, the role of ISG15 in HIV infection seems inconsistent. OseiKuffour et al. (2019) suggests depletion of ISG15 causes increasing misfolded p53 protein and further supports HIV replication. By contrast, depletion of ISG15 is considered to decrease susceptibility of HIV in CD4^+^T cells (Jurczyszak et al., 2022). Similarly, we identified significantly higher CD3+/CD45+ ratio in HIV patients with genotype AA and AT of rs77076061, which was also higher than the reference ratio. It suggested genotypes of rs77076061 in the *C19orf66* gene might affect immune response of the HIV patients in Yunnan. Thus, functions of ISGs in HIV infection, replication, and CD4^+^ T cells level of patients are needed further study.

The *C19orf66* gene is reported as IFN 1- induced ISG (Schoggins et al., 2011) and owns antiviral activity of abrogating DNA and RNA virus infection. C19orf66 interacts with programmed −1 ribosomal frameshifting (-1PRF) signal and causes premature translation termination, and further inhibits HIV replication (Wang et al., 2019). Because many viruses replicate by −1 PRF mechanism, C19orf66 is further identified to be a broad-spectrum inhibitor. Although the function of the *C19orf66* gene is to inhibit viral infection or replication, whether its genetic polymorphisms are associated with infectious disease is unknown. In this study, we firstly identify that genotypes of SNPs in the *C19orf66* gene are associated with HIV infection in Yunnan population. Furthermore, the relationship between SNPs and biochemical indices is found. In addition, the role of three SNPs in the *C19orf66* gene have been predicted in another study (Liu et al., 2023). In brief, three SNPs were located on the peak of H3K4me1, H3K4me3, and H3K27ac, which are considered as the promoter or enhancer regions and regulates the progress of gene transcription. Furthermore, dual-luciferase reporter assay was performed to verify the functional difference between two alleles of each SNP. Thus, we suggest C19orf66 could influence HIV infection and biochemical indices of HIV-infected persons in both functional and genetic aspects.

The frequencies of HIV-1 subtypes show difference between this study and the previous study. Zhang et al. (2006) collected 103, 015 HIV-1 infected persons to analyze the epidemiology in Yunnan. Similar to our results, they suggest that the epidemic subtypes are CRF07_BC, CRF08_BC, and CRF01_AE. However, the main subtype is CRF01_AE (occupies 40.5%) in Zhang et al. (2006), and only 18.15% of total HIV patients belongs to subtype CRF01_AE in this study. A few years later, Su et al. (2013) identified that CRF07_BC (18.9%), CRF08_BC (39.1%), and CRF01_AE (22.4%) are still the prevalent subtypes. However, the frequency of CRF01_AE decreased compared to Zhang et al. (2006). Recently, Chen et al. (2018) found the CRF08_BC was the main circulating recombinant form in Yunnan HIV population. In this study, the frequency of CRF07_BC and CRF 08_BC reaches 74.15%, and this result means the domain subtype of HIV-1 might gradually change in the past twenty years in Yunnan. We also found that CRF08_BC showed significant difference in HIV patients carried with various genotypes of rs12611087. This result might due to the prominently epidemic ratio of CRF08_BC. Although many studies identified the association between genetic polymorphisms and HIV-1 infection (Kim & Jeong, 2020), no study was performed in patients with different HIV-1 subtypes in Yunnan Province. Thus, we will further study the association between host genetic polymorphism and different subtypes of HIV-1 infection.

We firstly identify that the SNPs of the *C19orf66* gene are associated with HIV-1 infection in Yunnan population. The biochemical indices of HIV-1 patients are influenced by genotypes of rs77076061 in the *C19orf66* gene.

##  Supplemental Information

10.7717/peerj.16005/supp-1 Supplemental Information 1 Genotypes of each sample

## Additional Information and Declarations

### Data Availability

The following information was supplied regarding data availability: The raw data are available in the Supplementary File.
